# Involvement of TLR4 signaling regulated-COX2/PGE2 axis in liver fibrosis induced by *Schistosoma japonicum* infection

**DOI:** 10.1186/s13071-021-04790-7

**Published:** 2021-05-25

**Authors:** Lan Chen, Xiaofang Ji, Manni Wang, Xiaoyan Liao, Cuiying Liang, Juanjuan Tang, Zhencheng Wen, Ferrandon Dominique, Zi Li

**Affiliations:** 1 Sino‑French Hoffmann Institute, Guangzhou Medical University, Guangzhou, 511436 Guangdong People’s Republic of China; 2 Université de Strasbourg, M3I UPR9022 du CNRS, 67000 Strasbourg, France

**Keywords:** TLR4 signaling, COX2/PGE2 axis, Liver fibrosis, *Schistosoma japonicum*

## Abstract

**Background:**

Hepatic stellate cell (HSC) activation plays a pivotal role in hepatic inflammation and liver fibrosis. TLR4 pathway activation has been reported to be involved in mice liver fibrosis induced by hepatitis virus infection, alcohol abuse, biliary ligation, carbon tetrachloride 4 treatment, and *Schistosoma japonicum* (*Sj*) infection. The effect and mechanisms of the cyclooxygenase 2 (COX2)/prostanoid E2 (PGE2) axis on liver fibrosis induced by *Sj* are still unclear.

**Methods:**

Mice liver fibrosis were induced by cutaneous infection of *Sj* cercariae. COX-2 inhibitor, NS398 were injected from week 5 to week 7, while TLR4 inhibitor TAK242 were injected from week 4 to week 8 post *Sj* infection. Human HSCs line, LX-2 cells were cultured and exposed to LPS or synthetic PGE2, or pretreated by TAK242, TLR4-siRNA or NS398. Liver tissue and serum or in vitro cultured cell lysaste were collected at indicated time courses for exploring the relationship between TLR4 and COX2-PGE2 axis through qPCR, western blot, immunohistochemical assay, ect. One-way analysis of variance among multiple groups followed by Uncorrected Fisher’s LSD-*t* test or paired comparisons through *t* test were performed to tell the statistical differences.

**Results:**

This study investigated the link between the COX2/PGE2 axis and TLR4 signaling in the induction of liver fibrogenesis in mice during *Sj* infection and in vitro culture of HSC strain-LX-2. The COX2/PGE2 axis was positively associated with *Sj*-induced liver fibrosis. TLR4 pathway activation stimulated the COX2/PGE2 axis in *Sj-*infected mice and in lipopolysaccharide (LPS)-exposed cultured HSCs. Synthetic PGE2 activated cultured HSCs through upregulation of alpha smooth muscle actin (α-SMA) expression. In LPS-triggered HSCs, NS398, a COX2 inhibitor, led to suppression of PGE2 synthesis and reduced expression of α-SMA and type I collagen (COL I).

**Conclusions:**

These results indicate firstly the positive association of the COX2/PGE2 axis with liver fibrosis induced by *Sj* infection. TLR4 signaling may at least partially control the COX2/PGE2 axis in *Sj*-infected mice liver and in vitro cultured HSCs. The COX2/PGE2-EP2/EP4 axis might be a good drug target against liver fibrosis induced by *Sj* infection.

**Graphic abstract:**

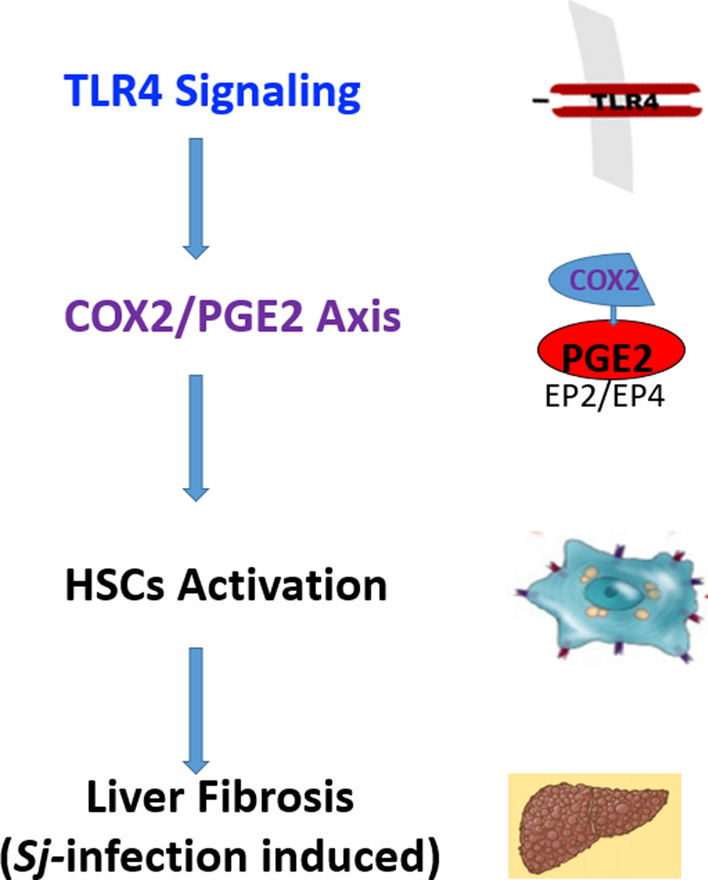

## Background

Prostaglandin E2 (PGE2) is produced when arachidonic acid is released from the plasma membrane and metabolized by two types of cyclooxygenases (COX), COX-1 and COX2. Three distinct PGE2 synthases (PGES) contribute specifically to PGE2 synthesis. Membrane-bound PGES-2 (mPGES-2) and cytosolic PGES-1 (cPGES-1) are constitutively expressed and functionally coupled to COX-1 for maintenance of basal levels of PGE2, while mPGES-1 is frequently induced concomitantly with COX2 by various pro-inflammatory stimuli, thereby generating a transient spike in PGE2 levels [1]. PGE2 is actively transported out of cells by multidrug resistance-associated protein 4 (MRP4) and enters the extracellular microenvironment, where PGE2 binds with four cognate EP receptors (EP1–EP4) and initiates diverse biological signaling pathways. Alternatively, PGE2 is transported into cells via the prostaglandin transporter (PGT) [2]. Via the PGE2 receptors EP2 and EP4 that trigger downstream cAMP signaling, PGE2 stimulates inflammation, pain, proliferation of certain cell types such as smooth muscular cells, plasticity, and cell injury [3, 4].

Activated but not quiescent hepatic stellate cells (HSCs) of mice express COX2 [5, 6]. Interestingly, the association of COX2 and its dependent PGE2-EP2/EP4 axis with fibrogenesis remains controversial. A selective COX2 inhibitor, NS-398, blocked the induction of α-SMA and PGE2 in a human HSC cell line, LI90 [5, 7]. The COX2 inhibitor SC-236 also lowered the extent of CCl4-induced rat liver fibrosis [8]. Celecoxib, another specific inhibitor of COX2, ameliorated rat liver fibrosis induced by thioacetamide (TAA) by decreasing intrahepatic and intestinal lipopolysaccharide (LPS) levels [9]. The COX2/PGE2 axis was found to be positively associated with choline-deficient/l-amino acid-deficient diet (CDAA)-, TAA-, and bile duct ligation (BDL)-induced rat liver fibrosis [10, 11] and liver cirrhosis in rats, mice, and patients [9, 12]. In contrast, all organs from CCl4-treated mice had elevated PGE2 levels [12], while the COX2/PGE2 axis protected against fibrosis in the lungs [13] and kidneys [14]. Several studies have also indicated that the COX2/PGE2 axis counteracted hepatic fibrogenesis by inhibiting the proliferation, contractility, and migration of HSCs and decreasing the production of ECM [15, 16]. *Schistosoma japonicum* (*Sj*) infection is still existent in 12 provinces in China. Infection of *Sj* leads to severe liver fibrosis induced by eggs trapped in the liver. The role and mechanisms of the COX2/PGE2 axis in *Sj*-induced liver fibrosis are still unclear.

Hepatic fibrosis is a pathophysiologic process commonly resulting from chronic liver injury and inflammation. HSCs transdifferentiate into alpha smooth muscle actin (α-SMA)-expressing myofibroblasts, which proliferate and secrete inflammatory cytokines and produce excessive extracellular matrix (ECM) including collagen type I (COL I) and type III (COL III). These three molecules are hallmarks of liver fibrosis [17]. TGF-β1 signaling activation is a well-known mechanism that mediates HSC activation [18]. TGF-β1 of parasitic origin was found to correlate with the extent of *Sj*-induced liver fibrosis [19]. Recent studies suggest that pathogen-associated molecular patterns (PAMPs), gut microflora-derived bacterial products such as LPS, bacterial DNA, and endogenous substances released from damaged cells, known as damage- or danger-associated molecular patterns (DAMPs), activate hepatic TLRs that contribute to the development of liver fibrosis [20]. Activated HSCs express high levels of TLR4 [21]. Reported mechanisms of TLR4 signaling-induced HSC activation and liver fibrosis include (1) BMP and activin membrane-bound inhibitor BAMBI downregulation, a TGF-β signaling inhibitor in quiescent HSCs [22, 23]; (2) miR-29 downregulation, which targets COL I expression [24]; (3) increased levels of LPS and DAMPs directly activating TLR4 signaling thereby activating nuclear factor κB (NF-κB) and c-Jun N-terminal kinase (JNK) that induce the production of many pro-inflammatory chemokines like monocyte chemoattractant protein-1 (MCP-1) and CCL-5, which in turn promote HSC activation [25, 26]. We have shown that the positive feedback regulation between transglutaminase 2 (TGM2) and TLR4 signaling in HSCs correlated with liver fibrosis after *Sj* infection [27].

TLR4 signaling controls the activation of the COX2-PGE2 pathway in macrophages [16, 28], intestinal epithelial cells [29], esophagus [30], and auditory cells [31]. Both TLR4 signaling and the COX2/PGE2 axis are involved in HSC activation and hepatic fibrosis. However, no research has reported whether there is a link between the COX2/PGE2 axis and TLR4 signaling pathway in HSC activation and fibrogenesis during *Sj* infection. Our work aimed to investigate the role of the COX2/PGE2 axis in *Sj*-induced liver fibrosis and to determine whether the relationship between it and TLR4 signaling is the mechanism.

## Methods

### Reagents

NS398, an inhibitor of COX2 activity, was obtained from MedChemExpress (HY-13913, New Jersey, USA). TAK242, a TLR4 inhibitor, was purchased from Shanghai Haoyuan Chemoexpress (Shanghai, China). TRIzol was obtained from Life Technologies (Waltham, MA, USA). SYBR^®^ Premix Ex Taq™ II (RR820A) and PrimeScript™ RT reagent kit with gDNA Eraser (RR047A) were purchased from TaKaRa Biotechnology Co. Ltd. (Dalian, China). The antibodies used were as follows: anti-COX2 (12282, Cell Signaling Technology, USA), anti-mPGES-1 (160140, Cayman, USA), anti-EP1 (ab217925, Abcam, USA), anti-EP2 (ab167171, Abcam, USA), anti-EP3 (SC-20676, Santa Cruz Biotechnology, USA), anti-EP4 (SC-55596, Santa Cruz Biotechnology, USA), anti-GAPDH (ab181603, Abcam, USA), anti-α-SMA (BS70000, Bioworld, USA), anti-COL I (BA0325, Boster Bio, USA), anti-TLR4 (ab47093, Abcam, USA), anti-p65 (6956, Cell Signaling Technology, USA), anti-NF-kB p65 (3033, Cell Signaling Technology, USA), anti-β-tubulin (DKM9003, Sanjian Biotechnology, China), and horseradish peroxidase (HRP)-conjugated secondary antibodies of mice or rabbit IgG (35552 and 35510, Invitrogen, Waltham, MA, USA). The bicinchoninic acid (BCA) protein assay kit was purchased from Guangzhou Dingguo Biotechnology (Guangzhou, China). Polyvinylidene fluoride (PVDF) membrane (ISEQ00010) was purchased from Merck Millipore (Darmstadt, Germany). The 3,3′-diaminobenzidine (DAB) substrate kit was purchased from Gene Tech Company Limited (Shanghai, China). Enhanced chemiluminescent (ECL) reagent was obtained from GBCBIO Technologies, Guangzhou, China. Sirius red staining (connective tissue staining) kit was purchased from Abcam (ab150681, USA). LPS and synthetic PGE2 were obtained from Sigma-Aldrich (USA), Dulbecco's modified Eagle medium (DMEM) from Thermo Fisher (Gibco), and fetal bovine serum (FBS) from ExCell Bio. LPS levels in sera were detected using a competitive enzyme-linked immunosorbent assay kit for LPS from antibodies-online.com (ABIN6574100, USA). The Ribo FECT CP Transfection Kit was purchased from RiboBio Biotechnology Limited (C10511, Guangzhou, China).

### Mice, parasite infection, and treatments

Six- to eight-week-old female C57BL/6 mice were obtained from SPF Biotechnology Co. Ltd (Beijing) and maintained according to institutional guidelines. All mice experiments were approved as appropriate and humane by the Institutional Animal Care and Use Committee at South China Agricultural University. Mice were infected percutaneously through the abdomen with 20 ± 3 *Sj* cercariae of the Chinese mainland strain. From week 5 to week 7 after *Sj* infection, mice were treated with NS398 (3 mg/kg body weight) in 100 μl 2% DMSO by intraperitoneal injection 3 times a week (*n* = 8), while the infection mice group only received 2% DMSO (*n* = 7). Two non-infected control mice groups were treated with NS398 (*n* = 5) and 2% DMSO (*n* = 9), respectively. Elsewhere, mice at 4-weeks post-infection were injected with 100 μl TAK242 (0.3 mg/kg body weight) dissolved in PBS through the intraperitoneal route twice a week for 4 weeks (*n* = 8), while the infection control group only received PBS (*n* = 7). Two non-infected control mice groups were treated with TAK242 (*n* = 7) and PBS (*n* = 7), respectively. At indicated time courses, mice were sacrificed and samples were collected for further analysis.

### RNA isolation and quantitative reverse transcription PCR

Total RNA was extracted from fresh mice livers using TRIzol reagent. Genomic DNA was removed, and then cDNA was synthesized using the PrimeScript™ RT reagent kit with gDNA Eraser. The relative RNA expression level of target genes was measured by real-time quantitative reverse transcription polymerase chain reaction (RT-qPCR) with the SYBR^®^ Premix Ex Taq™ II kit and the CFX96™ real-time system according to the manufacturer’s instructions. The RT-qPCR primer pairs are listed in Table 1. The RNA expression level of each gene was normalized to GAPDH and analyzed using the 2^−ΔΔCt^ data analysis method.

**Table 1:** Primer pair sequences of mice genes used in RT-qPCR

Proteins	Genes	Forward primer sequence	Reverse primer sequence
GAPDH	*Gapdh*	TGTGTCCGTCGTGGATCTGA	TTGCTGTTGAAGTCGCAGGAG
COX2	*COX2*	TTCCAATCCATGTCAAAACCGT	AGTCCGGGTACAGTCACACTT
mPGES-1	*Ptges*	CACACTGCTGGTCATCAAGAT	TCACTCCTGTAATACTGGAGGC
EP1	*Ptger1*	TGCTTGCCATCGACCTAGC	CACCCAGGAAATGACACGC
EP2	*Ptger2*	CAGCTCGGTGATGTTCTCGG	GAGCACCAATTCCGTTACCAG
EP3	*Ptger3*	CAGCTCATGGGGATCATGTGT	CTCAACCGACATCTGATTGAAGA
EP4	*Ptger4*	CTTGTTGGTAAGCCCGGTGA	AGACCCGACAGACCGAAGAA
MRP4	*Abcc4*	GGCACTCCGGTTAAGTAACTC	TGTCACTTGGTCGAATTTGTTCA
PGT	*Slco2a1*	CGACTCCTCCTGTATCCGGT	TGTTCTTCTTCACCCTCCAGC

GAPDH and *Gapdh*: glyceraldehyde-3 phosphate dehydrogenase; COX: cyclooxygenase; mPGES-1: microsomal prostaglandin E synthase-1; Ptges: prostaglandin E synthase; EP1 and Ptger1: prostaglandin E receptor 1; MRP4: multidrug resistance-associated protein 4; *Abcc4*: ATP binding cassette subfamily C member 4; PGT: prostaglandin transporter; *Slco2a1*: solute carrier organic anion transporter family member 2A1

### Sirius red staining

Fresh hepatic tissues were fixed in 4% paraformaldehyde for 24 h, and then embedded with paraffin. Four-micrometer liver sections were prepared and stained with Sirius red to semi-quantify the extent of collagen deposition, which demonstrates the severity of liver fibrosis. The collagen deposition area of all granulomas around *Sj* single eggs as displayed in deep red was measured and analyzed by ImageJ software. Each section was evaluated in double-blind fashion by two independent researchers.

### Western blotting

The proteins from cultured LX-2 cells or mice liver tissue were extracted using radio-immunoprecipitation assay buffer (RIPA, P0013B, Beyotime, Shanghai, China) and quantified using a BCA protein assay kit (23227, Thermo Fisher Scientific, USA). Equal quantities of total protein lysate were resolved on 10% sodium dodecyl sulphate (SDS)–polyacrylamide gels and then transferred to PVDF membranes. After incubation with the indicated primary and secondary antibodies, the target proteins were visualized using an ECL reagent. The intensity of the individual bands was semi-quantified by ImageJ and normalized to the corresponding input control (GAPDH or β-tubulin) bands. Fold changes were calculated with the control taken as 1.

### Immunohistochemical assay

Endogenous peroxidase in mouse liver sections was blocked with 3% hydrogen peroxide (H_2_O_2_). Immunohistochemical (IHC) staining assay was used to determine the expression level and location of α-SMA and COL I in the mice liver tissue using anti-α-SMA (1:200) and anti-COLI (1:400) primary antibodies, followed by HRP-conjugated secondary anti-rabbit or anti-mouse antibodies. The images of 5 fields of every section in each mouse of every group were observed and captured with an optical microscope equipped with a camera (Olympus, Tokyo, Japan). The semi-quantitative analysis was determined by imageJ software [27].

### Cell culture and treatment

A human HSC line, LX-2, was cultured in DMEM with 10% fetal bovine serum (FBS). When confluence reached 80–90%, LX-2 cells were exposed to LPS or synthetic PGE2, or these cells were pretreated by TAK242 for 30 min or NS398 for 15 min at the indicated concentrations prior to stimulation with LPS.

### RNA interference

LX-2 cells were seeded in a six-well plate the day before transfection at 30–50% confluence. TLR4-specific small interfering RNAs (siRNA), or negative control siRNAs, synthesized by Ribobio Biotechnology Limited, were transfected using the Ribo FECT CP Transfection Kit according to the manufacturer’s instructions. Twenty-four hours after transfection, cells were treated with LPS or mock-treated for 24 h. The total protein of cells in RIPA lysis buffer with phenylmethylsulfonyl fluoride (PMSF) was then extracted to be measured by western blotting. TLR4 siRNA sequences are as follows: TLR4 siRNA-1, 5′-GGACAACCAGCCTAAAGTA-3′; TLR4 siRNA-2, 5′-GGTGTGAAATCCAGACAAT-3′.

### Detection of serum LPS levels

Serum LPS was quantitatively detected by competitive inhibition enzyme immunoassay. An antibody specific to LPS was pre-coated onto the microtiter plate. A competitive inhibition reaction was launched between biotin-labeled LPS and unlabeled LPS (standards or samples) with the pre-coated antibody specific to LPS. After incubation, the unbound conjugate was washed off. Next, avidin conjugated to HRP was added to each microtiter plate well and incubated. The amount of bound HRP conjugate is inversely proportional to the concentration of target in the sample. After the addition of the substrate solution, the intensity of color developed is inversely proportional to the concentration of target in the sample. The detailed protocol is shown in https://product-manuals.abocdn.com/ABIN6574100.pdf.

### Detection of serum ALB concentration

The concentration of serum albumin (ALB) was measured via the bromocresol green colorimetric method using an automatic analytical instrument (cobas^®^ 8000 Modular Analyzer Series; Roche Group, Switzerland). Briefly, in an acidic condition, ALB combines with negatively charged bromocresol green to form a blue–green complex with an absorption wave at 628 nm. The absorbance of the complex was proportional to the concentration of ALB.

### Statistical analysis

The results are presented as the standard error of the mean (± SEM) of the indicated number of replicates/experiments. To calculate the statistical differences among multiple groups, we performed one-way analysis of variance (ANOVA) followed by Dunnett’s multiple comparisons test if the ANOVA result was significant. To determine the difference between two groups, we used unpaired *t* test analysis. An adjusted *p* value of ≤ 0.05 was considered statistically significant.

## Results

### The COX2/PGE2 axis is correlated with Sj-induced liver fibrosis

**Fig. 1:**
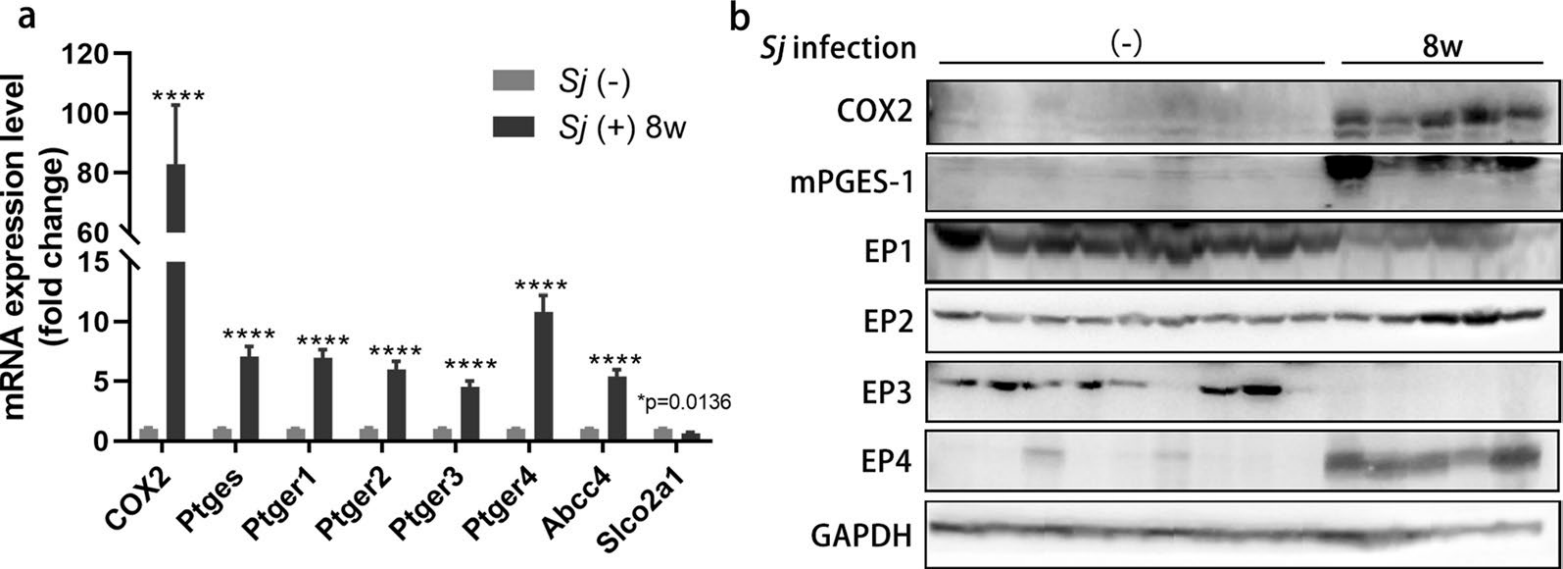
The components of the COX2/PGE2 axis are overexpressed in the livers of mice with 8-week *Sj* infections. C57BL/6 mice were infected with 20 ± 3 cercariae of *Sj* for 0 or 8 weeks. Mice liver samples were kept in TRIzol or in RIPA for RNA or total protein extraction respectively. **a** The relative RNA expression level of *COX2*,* Ptges*, *Ptger1-4*, *Abcc4*, and *Slco2a1* in indicated mice liver were measured using RT-qPCR. *Gapdh* acts as an internal control (*t* test, *p < 0.05, ****P < 0.0001). **b** The protein expression level of COX2, mPGES-1, and EP1-4 in mice liver homogenates were detected by western blotting. GAPDH is used as a loading control. Data are presented as mean ± SEM from 5 to 9 mice per group

Our previous studies have shown that the extent of hepatic fibrosis reached advanced levels at week 8 of *Sj* infection [27, 32]. To evaluate the correlation between the COX2/PGE2 axis and hepatic fibrosis, the transcriptional levels of *COX2*, *Ptges*, *Ptger1-4*, *Abcc4*, and *Slco2a1* in mice liver were measured using RT-qPCR (Fig. 1a), and the translational levels of COX2, mPGES-1, and EP1-4 in mice liver were detected by western blotting (Fig. 1b) in 8-week *Sj*-infected and uninfected mice groups. Compared with the uninfected mice, the RNA expression levels of *COX2*, *Ptges*, *Ptger1-4*, and *Abcc4* were significantly increased in the *Sj*-infected mice, while *Slco2a1* was significantly decreased (*t* test: week 8 vs. (−): COX2: *t*_(12)_ = 5.690, *P* = 0.0001; *Ptges*: *t*_(12)_ = 9.702, *P* < 0.0001; *Ptger1:* *t*_(12)_ = 12.02, *P* < 0.0001; *Ptger2:* *t*_(12)_ = 9.783, *p* < 0.0001; *Ptger3:* *t*_(12)_ = 8.954, *P* < 0.0001; *Ptger4:* *t*_(12)_ = 9.550, *P* < 0.0001; *Abcc4:* *t*_(12)_ = 10.58, *p* < 0.0001; *Slco2a1*:* t*_(12)_ = 2.888, *P* = 0.0136). Protein expression levels of COX2, mPGES-1 and EP2, EP4 were also increased in week 8 *Sj*-infected mice.

**Fig. 2:**
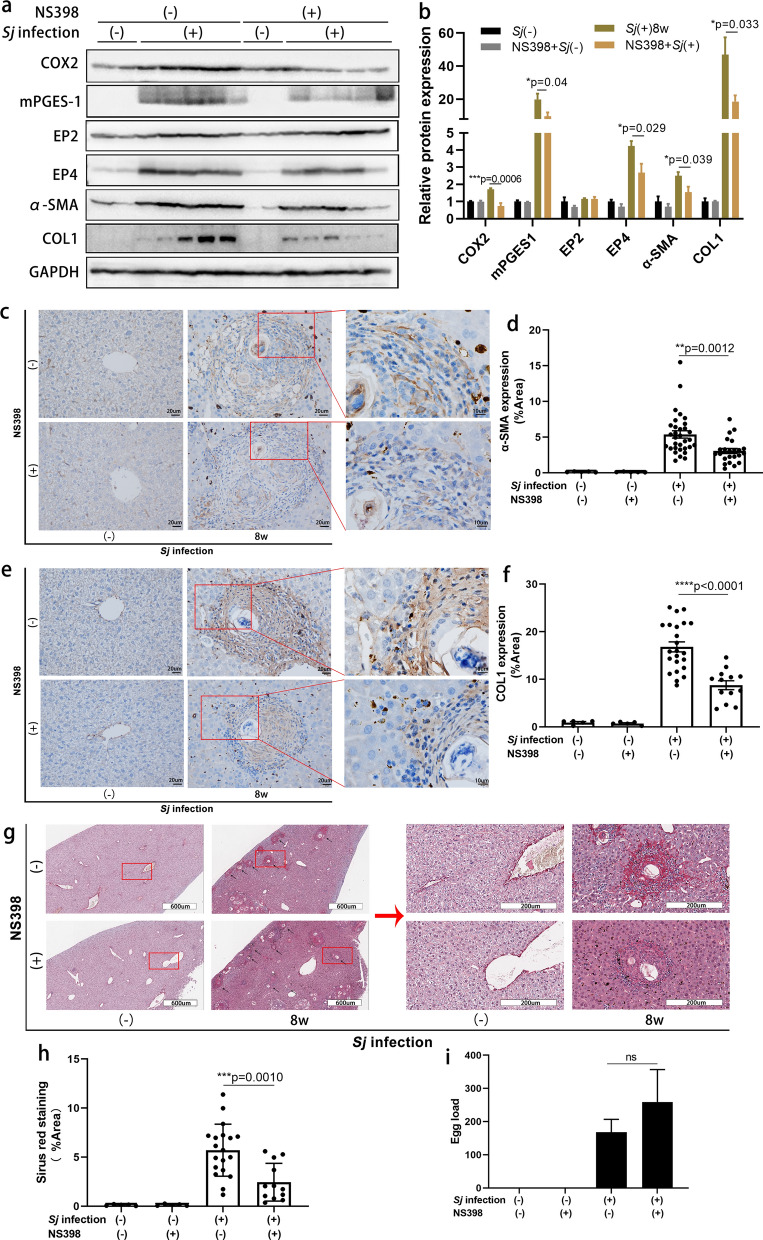
Suppression of COX2 activity with NS398 consistently diminished the expression level of the components of the COX2/PGE2 axis and the extent of hepatic fibrosis. The activity of COX2 in mice was inhibited by NS398 through intraperitoneal injection three times per week from week 5 to week 7 after *Sj* infection. Mice were sacrificed at week 8. Non-infected mice with or without NS398 treatment served as controls. **a**, **b** The protein expression level of COX2, mPGES-1, EP2, EP4 and α-SMA, COL I in mice liver homogenates detected by western blotting was displayed in **a**, and the semi-quantitative result is shown in **b** (*t* test, **p < 0.01, ***P < 0.001**)**. GAPHD was used as a loading control. **c**–**f** The protein expression levels and location of α-SMA (**c**) and COL I (**e**) in mice liver tissue were determined by IHC assay (×200), and the semi-quantitative result for these proteins is shown using ImageJ analysis in **d** (*t* test, **p < 0.01) and **f** (*t* test, ****P < 0.0001). Representative Sirius red staining (×200) of *Sj* single egg granuloma is shown in **g**. The percentage of morphometric collagen areas of single *Sj* egg granuloma is shown in **h** **(***t* test, ***P < 0.001). **i** The average number of *Sj* eggs on each liver section is shown (*t* test, ns, no significance). Data are presented as mean ± SEM from 5 to 9 mice per group

To assess whether the COX2/PGE2 axis is involved in the formation of liver fibrosis induced by *Sj* infection, we injected *Sj*-infected mice with the COX2 inhibitor NS398 beginning week 5 of infection. The whole treatment lasted for 3 weeks. NS398 treatment lowered the protein expression levels of COX2, mPGES-1, EP4 and α-SMA, COL I according to western blotting results (Fig. 2a, b) (*t* test: DMSO/*Sj*(+) vs. NS398/*Sj*(+): COX2: *t*_(8)_ = 5.409, *P* = 0.0006; mPGES-1: *t*_(8)_ = 2.450, *P* = 0.0399; EP4: *t*_(8)_ = 2.660, *P* = 0.0288; α-SMA: *t*_(8)_ = 2.453, *P* = 0.0397; COLI: *t*_(8)_ = 2.569, *P* = 0.0332), without a significant change on EP2 (*t* test: DMSO/*Sj*(+) vs. NS398/*Sj*(+): *t*_(8)_ = 0.8025, *P* = 0.9534) . The expression of α-SMA changed from moderate to low levels and COL I from high to moderate levels according to results evaluated by ImageJ. In the IHC assay (Fig. 2c–f) (*t* test: DMSO/*Sj*(+) vs. NS398/*Sj*(+): α-SMA: *t*_(53)_ = 3.437, *P* = 0.0012; COLI: *t*_(34)_ = 5.107, *P* < 0.0001). In addition, the NS398-treated mice exhibited a significant reduction in the extent of collagen deposition after *Sj* infection (Fig. 2g, 2h) (*t* test: DMSO/*Sj*(+) vs. NS398/*Sj*(+): *t*_(29)_ = 3.681, *P* = 0.0010). The average *Sj* egg load per liver section showed a tendency to increase, but the difference was not significant (Fig. 2i) (*t* test: DMSO/*Sj*(+) vs. NS398/*Sj*(+): *t*(6) = 0.8619, *P* = 0.4218). This indicated that the COX2/PGE2 axis is not only positively associated with CDAA-, TAA-, and BDL-induced rat liver fibrosis [10, 11] and liver cirrhosis in rats, mice, and patients [9, 12], but also positively related to *Sj* infection-induced liver fibrosis.

### TLR4 pathway activation stimulated the COX2/PGE2 axis in Sj-infected mice and in LPS-exposed cultured HSCs

**Fig. 3:**
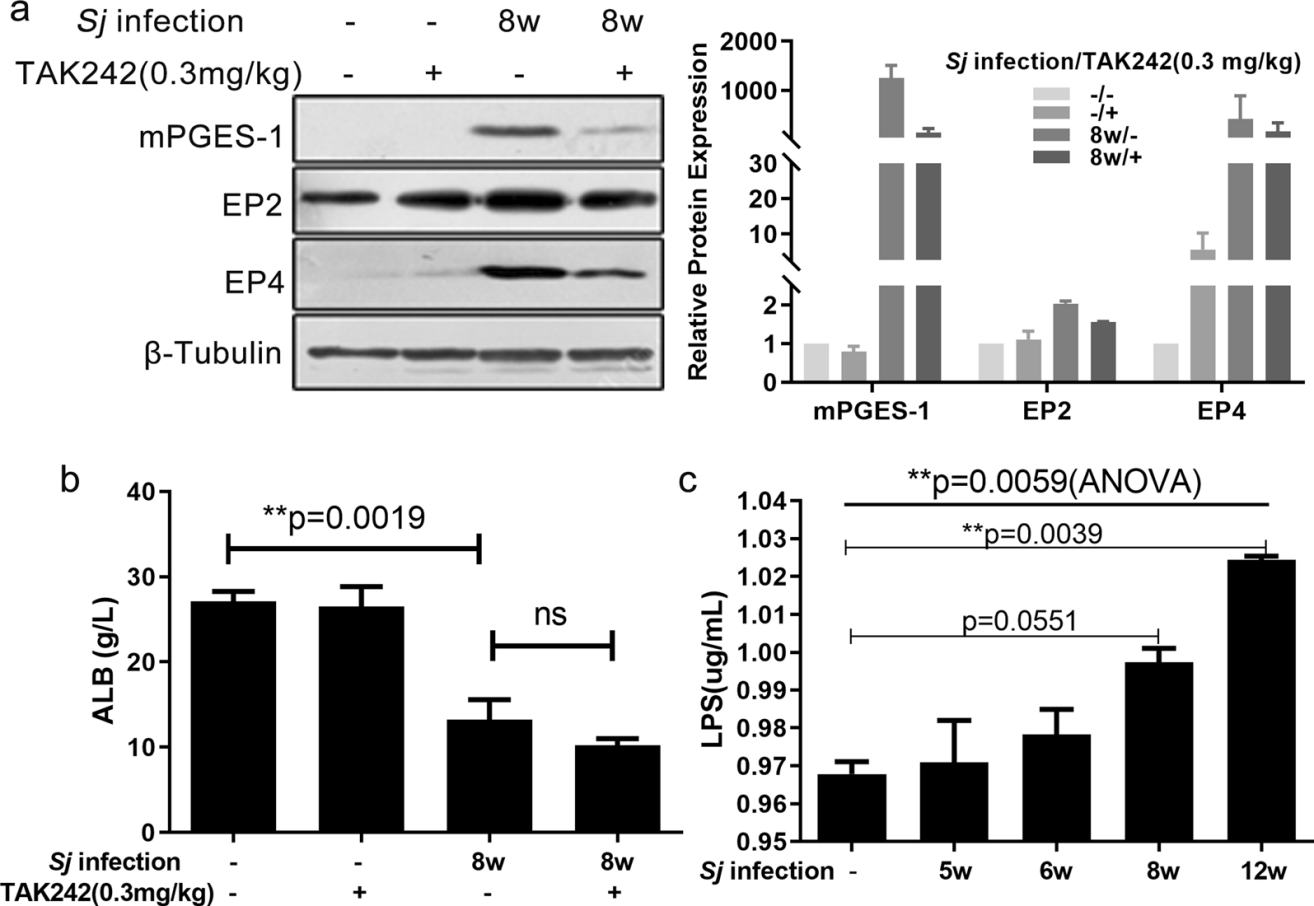
TLR4 pathway activation stimulated the COX2/PGE2 axis in *Sj*-infected mice. TLR4 signaling in C57BL/6 mice was inhibited by intraperitoneal injection of TAK242 once a day from weeks 4 to 8 after *Sj* infection. **a** The protein expression level of mPGES-1, EP2, and EP4 in mice liver homogenates tested by western blotting is shown on the left, and semi-quantitative results are displayed on the right. **b** Concentration of serum ALB in TAK242-treated or untreated mice with or without *Sj* infection was detected by the bromocresol green colorimetric method (*t* test, **P < 0.01, ns, no significance). **c** Concentration of LPS in sera of mice with indicated time courses was detected by ELISA (ordinary one-way ANOVA followed by Dunnett’s multiple comparisons test, **P < 0.01). Data are presented as mean ± SEM from 7 to 10 mice per group. All experiments were performed twice

TLR4 signaling has been reported to induce HSC activation and liver fibrosis through many mechanisms [22, 23]. The relationship between TLR4 and the COX2/PGE2 axis in the *Sj*-infected liver is still unknown. To determine whether the TLR4 pathway regulates the COX2/PGE2 axis during *Sj* infection-induced liver fibrosis, we examined the levels of several key proteins in this axis using infected mice liver after TAK242 treatment. TAK242 treatment downregulated the expression of mPGES-1, EP2, and EP4 in infected mice liver (Fig. 3a). Albumin (ALB) has been reported to trigger PGE2 degradation [9, 30]. Here, the level of serum ALB was significantly decreased in the 8-week *Sj*-infected group compared to the non-infected group, and TAK242 treatment did not reverse this reduction (Fig. 3b) (*t* test, *Sj*(+) vs.* Sj*(−):* t*_(5)_ = 5.932, *P* = 0.0019). This observation rules out an implication of albumin in modulating PGE2 levels as a result of TLR4 pathway inhibition. Because *Sj* egg deposition increased the richness of microbiome in the gut of infected mice [33], we checked the level of LPS in serum, which might increase as a result from the larger quantities of Gram-negative bacteria in the gut. The concentration of serum LPS slightly increased gradually with *Sj* infection (Fig. 3c) [ordinary one-way ANOVA, *F*_(4, 5)_ = 14.43, *P* = 0.0059, followed by Dunnett’s multiple comparisons test: week 5 vs. (−): *P >* 0.9999, week 6 vs. (−): *P* > 0.9999, week 8 vs. (−): *P* = 0.0551, week 12 vs. (−): *P* = 0.0039], likely to a too limited extent to produce a significant biological response.

**Fig. 4:**
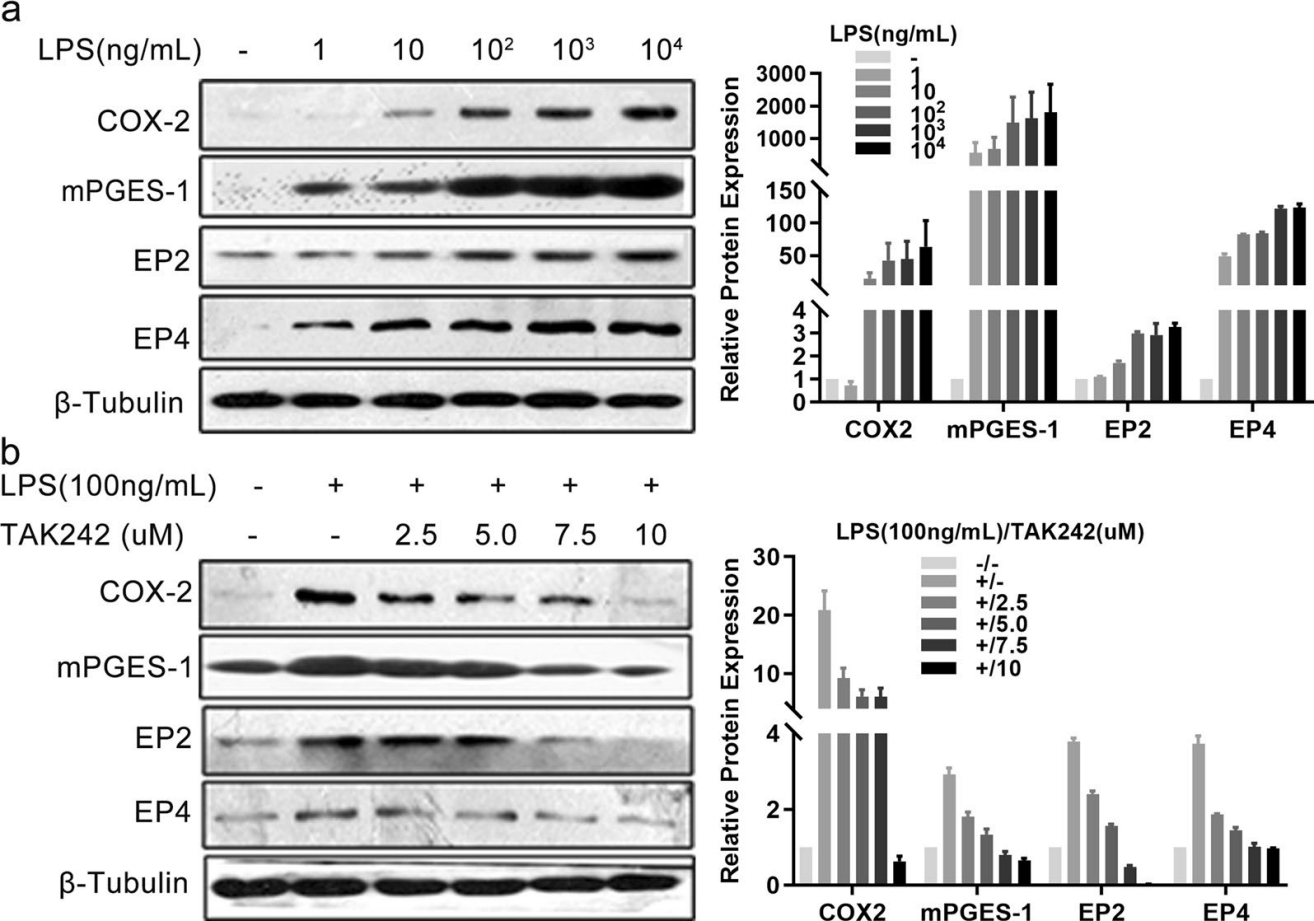
TLR4 pathway activation stimulated the COX2/PGE2 axis in cultured HSCs. **a** Cells of the hepatic stellate cell line LX-2 were triggered with indicated concentrations of LPS for 24 h. The protein expression level of COX2, mPGES-1, EP2, and EP4 detected by western blotting is displayed in the left panel, and the semi-quantitative result is shown in the right panel. **b** LX-2 cells were pretreated with TAK242 for indicated concentrations for 30 min prior to stimulation with LPS (100 ng/ml) for 24 h. Non-treatment or LPS (100 ng/ml) treatment alone were used as controls. Indicated protein expression levels were evaluated by western blotting as shown in the left panel, and the semi-quantitative result is shown in the right panel. β-Tubulin was used as a loading control. Each experiment was conducted two or three times

To validate whether the TLR4 pathway regulates the COX2/PGE2 axis in the human HSC line LX-2, we examined the levels of several key proteins in this axis using cells exposed to LPS alone or after TAK242 treatment. COX2, mPGES-1, EP2, and EP4 were distinctly induced by LPS in a dose-dependent manner (Fig. 4a), and TAK242 pre-treatment gradually lowered these inductions, especially at a dose of 10 µM (Fig. 4b).

In summary, our data are compatible with the model, according to which TLR4 pathway activation stimulates the COX2/PGE2 axis in *Sj-*infected mice and in LPS-exposed cultured HSCs.

### Verification of the relationship between TLR4 signaling and HSC activation and fibrogenesis

**Fig. 5:**
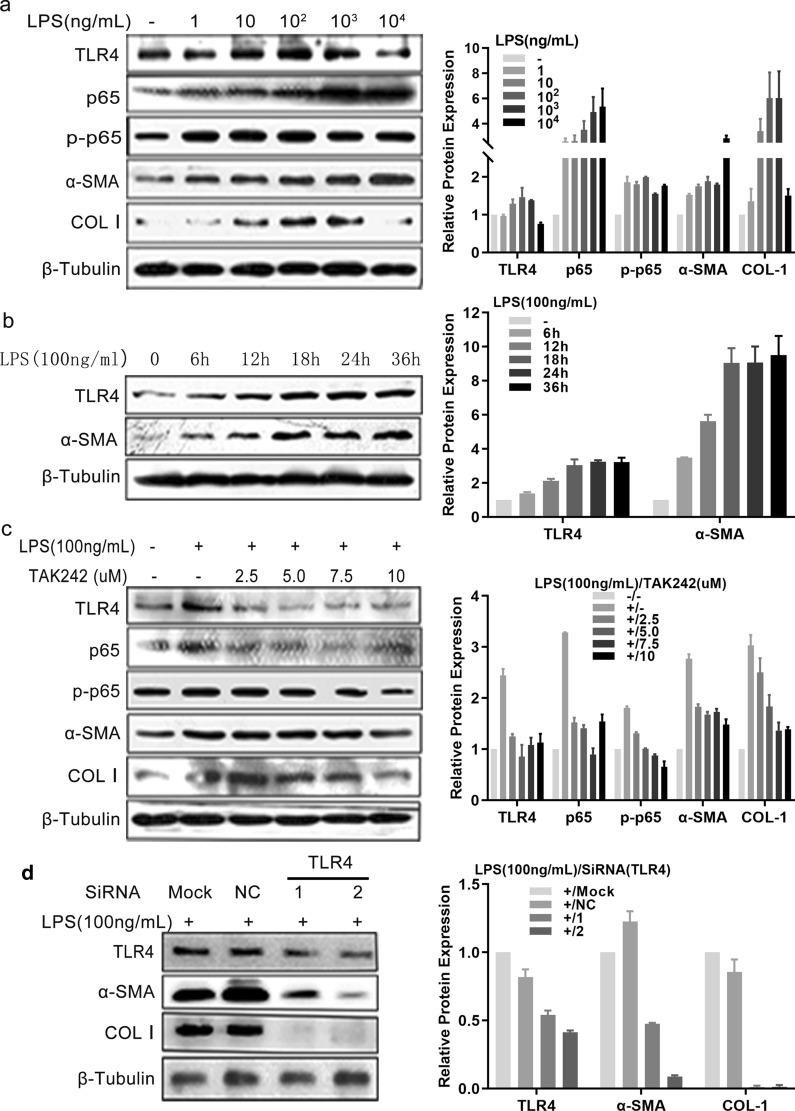
TLR4 signaling was verified to induce hepatic stellate cell activation and fibrogenesis in vitro. **a** LX-2 was triggered with different doses of LPS for 24 h or was triggered with LPS (100 ng/ml) for indicated time courses. **b**, **c** LX-2 cells were pretreated with TAK242 for indicated concentrations for 30 min prior to stimulation with LPS (100 ng/ml) for 24 h. Non-treatment or LPS treatment alone were used as controls. **d** LX-2 cells were transfected with either control siRNA (NC) or two different TLR4 siRNAs and cultured for 24 h, and then stimulated with LPS (100 ng/ml) for 24 h. Indicated protein expression levels were determined using western blotting as shown in the left panels, and the semi-quantitative results are shown in the right panels. Each experiment was conducted at least twice. β-Tubulin was used as a loading control

To verify the correlation between TLR4 signaling and HSC activation and fibrogenesis, the LX-2 HSCs were employed and protein expression levels were monitored after exposure to LPS, or treatment with TAK242 or TLR4-specific siRNAs*.* LPS treatment from 1 ng/ml to 1 µg/ml enhanced TLR4 signaling as monitored by the increased expression levels of TLR4 and p-p65, and the upregulated protein expression of α-SMA and COL I (Fig. 5a). The protein levels of TLR4 and α-SMA in LPS-treated LX-2 (100 ng/ml) increased with the time of incubation (Fig. 5b). The increased TLR4, p-p65, α-SMA and COL I levels after LPS stimulation were reduced by TAK242 treatment, especially at a dose of 10 µM (Fig. 5c). The upregulation of α-SMA and COL I induced by LPS treatment was markedly decreased after TLR4-specific siRNA transfection (Fig. 5d). These data verified that TLR4 signaling is positively associated with HSC activation and fibrogenesis.

### The COX2/PGE2 axis was required for TLR4 signaling-induced HSC activation and fibrogenesis

**Fig. 6:**
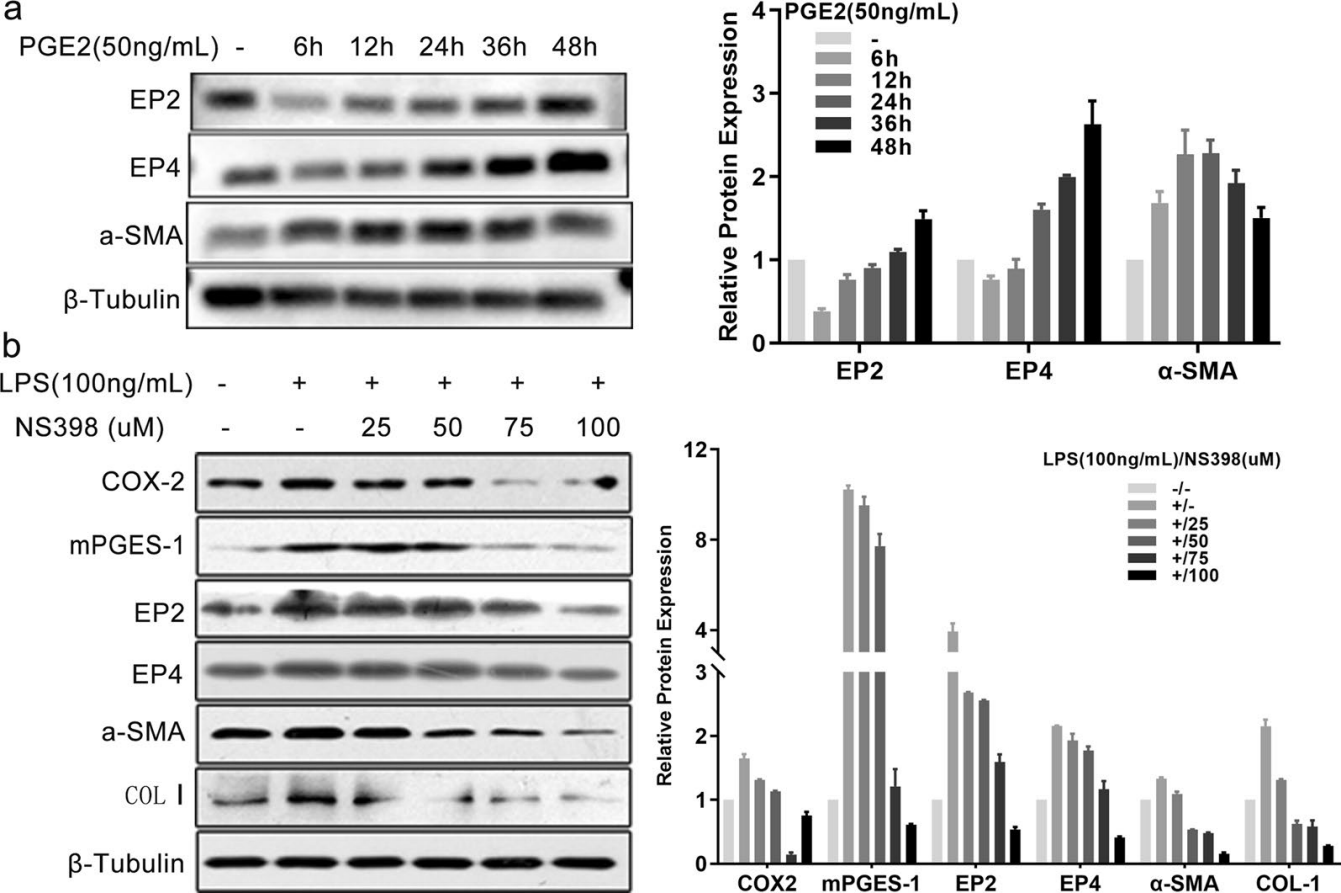
COX2/PGE2 axis induced LPS-mediated HSC activation and fibrogenesis in vitro. **a** LX-2 cells were triggered with synthetic PGE2 (50 ng/ml) for the indicated times. **b** LX-2 cells were pretreated with indicated concentrations of NS398 for 15 min prior to stimulation with LPS (100 ng/ml) for 24 h. Non-treatment or LPS (100 ng/ml) treatment alone were used as controls. Indicated protein expression levels were evaluated by western blotting as shown on the left panels, and the semi-quantitative results are shown on the right panels. β-Tubulin was used as a loading control. Each experiment was conducted two or three times

To assess the role of PGE2 in HSC activation, we treated the LX-2 cell line with synthetic PGE2. HSC activation was enhanced by synthetic PGE2 as monitored by the augmentation of α-SMA protein expression, while the expression of both EP2 and EP4 increased with the time of incubation (Fig. 6a). As reported in macrophages, intestinal epithelial cells, esophageal, and auditory cells [28–31, 34], TLR4 signaling controls the activation of the COX2-PGE2 pathway. Herein, we observed that in cultured HSCs, the activation of TLR4 signaling by LPS increased the protein expression levels of COX2, mPGES-1, EP2, EP4, α-SMA, and COL I. These increased expression levels were reduced by NS398 treatment, especially at doses of 7.5 µM and 10 µM (Fig. 6b). Therefore, TLR4 signaling-dependent HSC activation and fibrogenesis is mediated at least partially through the COX2/PGE2 axis.

## Discussion

In this study, we investigated the relationship between the COX2/PGE2 axis and TLR4 signaling in the induction of liver fibrosis in mice during *Sj* infection and in vitro cultured HSCs. The COX2/PGE2 axis showed a positive association with the extent of liver fibrosis induced by *Sj* infection. TLR4 pathway activation stimulated the COX2/PGE2 axis in *Sj-*infected mice liver tissue and in cultured HSCs. TLR4 signaling was found to induce HSC activation and fibrogenesis in vitro, and the COX2/PGE2 axis mediated this function. This indicated that liver fibrosis was induced by *Sj* infection through the activation of TLR4 signaling and then the COX2/PGE2 axis.

Chronic liver inflammation leads to fibrosis and cirrhosis. Understanding the mechanisms of liver inflammation and fibrosis is critically important to develop treatments for liver diseases. Many reports have indicated that the COX2/PGE2 axis is positively associated with hepatic fibrogenesis [7–12, 33]. Nonsteroidal anti-inflammatory drugs inhibited the activity of COX and attenuated HSC proliferation, contractility, and migration, thereby alleviating fibrogenesis [10]. Efsen et al. demonstrated that NS398 inhibited MCP-1 expression via the prostaglandin-cAMP pathway in TNF-α- and IL-1β-stimulated HSCs [5, 35]. However, Hui et al. [15] suggested that the COX2/PGE2 axis inhibits both basal and TGF-β1-mediated induction of collagen synthesis by another HSC cell line, LX-1, and primary HSCs. In a mouse model of CCl4- and BDL-induced liver fibrosis, COX2 expression in hepatocytes induced apoptosis of HSCs and attenuated liver fibrosis through PGE2 by downregulating miR-23a-5p and miR-28a-5p [36]. Schippers et al. showed that PGE2 exerts anti-fibrotic activity by modulating cAMP effector Epac-1 production in HSCs, and a COX2 inhibitor, niflumic acid, effectively enhanced mice liver fibrosis induced by CCL4 [37]. Herein, we showed that the components of the COX2/PGE2 axis were significantly upregulated in *Sj*-infected mice liver and in LPS-triggered HSCs. NS398 effectively attenuated both PGE2 synthesis and protein production of α-SMA and COL I. In the *Sj*-infected mice liver, increased expression of COX-2 and mPGES-1 induced PGE2 synthesis, and high transcriptional levels of MRP4 and low levels of PGT also favored high levels of PGE2 in the liver microenvironment. The biological effects of PGE2 are mediated by EP1–EP4. EP2 and EP4, but not EP1 or EP3, showed high protein expression levels in the *Sj*-infected mice liver, which suggests that PGE2 induces liver fibrosis through EP2 and EP4.

We previously showed that TLR4 signaling in HSCs correlated with liver fibrosis after *Sj* infection [27]. As reported, TLR4 signaling controls the activation of the COX2/PGE2 axis in several cell types [30, 31, 34, 38, 39]. The NF-κB pathway, activated by LPS-triggered TLR4 signaling, contributes to the early production of prostaglandins through the expression of COX2 and mPGES-1 in the macrophages [27]. In the intestine, TLR4 signaling-mediated COX2 pathway activation through EP2 or EP4 was associated with intestinal epithelial cell proliferation and protection against apoptosis [34]. Our results showed that TLR4 pathway activation controls the expression of COX2, EP2/EP4, and synthesis of PGE2 in HSCs. Importantly, lower expression levels of mPGES-1 and EP2/EP4 were detected in *Sj*-infected mice liver with TAK242 treatment. Serum albumin does not appear to affect PGE2 levels in a TLR4-dependent manner. Our results taken in the light of the previous work suggest that TLR4 pathway activation stimulated the COX2/PGE2 axis in *Sj*-infected mice and in LPS-exposed cultured HSCs.

The current understanding of the role of the COX2/PGE2 axis in liver fibrosis is limited by the varied effects observed depending on the model considered and the use of diverse PGE2 synthesis inhibitors. In this study, although the extent of liver fibrosis induced by *Sj* infection was strikingly reduced by NS398 treatment, the egg load showed a tendency to increase. Moreover, *Sj*-infected mice with NS398 or TAK242 treatment exhibited manifestations as fatigue and in appetite. Three mice died during *Sj* infection and NS398 treatment, while only two mice died during *Sj* infection without NS398 treatment. This suggests that TLR4 signaling and the COX2/PGE2 axis may be required to protect against live *Sj* eggs. The medication time and dosage of these two inhibitors need to be carefully considered. Here, we treated mice with NS398 at a median dosage from week 5, when the granulomatous inflammation had existed for more than 1 week, until week 8. Whether drug treatment in the late phase will obtain better effects needs further study. Nevertheless, the anti-fibrotic effect of this drug in *Sj*-infected mice in this study is affirmative.

In this study, we confirmed that TLR4 signaling pathway activation in HSCs was required for their activation and fibrogenesis, not only during *Sj* infection. The induction of endogenous PGE2 synthesis by LPS-triggered TLR4 pathway activation might play a role in HSC activation, since synthetic PGE2 induced α-SMA expression in LX-2 cells. A high level of LPS-triggered TLR4 pathway activation and *Sj* infection positively controlled the COX2/PGE2 axis and subsequent HSC activation and fibrogenesis. NS398 was an effective inhibitor of this axis to ameliorate liver fibrosis induced by TLR4 pathway activation and *Sj* infection. The study of the mechanisms downstream of PGE2-EP2/EP4 signaling will be a worthy future pursuit.

## Conclusions

Our study firstly demonstrates the reciprocal relationships between TLR4 signaling and the COX2/PGE2 axis and the resulting HSC activation toward a fibrogenic phenotype. We also provide evidence of TLR4 signaling activation of the COX2/PGE2 axis that might contribute to mice liver fibrosis induced by *Sj* infection. The regulation of TLR4 signaling and COX2/PGE2 with a consequent decrease in liver fibrosis may represent a potential therapeutic approach for hepatic granuloma caused by *Sj* infection.

## Data Availability

The datasets supporting the conclusions of this article are included within the article.

## Abbreviations

HSCs: Hepatic stellate cells
*Sj*: *Schistosoma japonicum*
COX: Cyclooxygenase
PGE2: Prostanoid E2
α-SMA: Alpha smooth muscle actin
COLI: Collagen type I
TLR4: Toll-like receptor 4
PGES: PGE2 synthases
mPGES: Membrane-bound PGES
cPGES-1: Cytosolic PGES-1
LPS: Lipopolysaccharide
NF-κB: Nuclear factor kappa B
TAA: Thioacetamide
BDL: Bile duct ligation
ECM: Extracellular matrix

## References

[1] Nakanishi M, Rosenberg DW (2013). Multifaceted roles of PGE2 in inflammation and cancer. Semin Immunopathol.

[2] Kochel TJ, Goloubeva OG, Fulton AM (2016). Upregulation of cyclooxygenase-2/prostaglandin E2 (COX-2/PGE2) pathway member multiple drug resistance-associated protein 4 (MRP4) and downregulation of prostaglandin transporter (PGT) and 15-prostaglandin dehydrogenase (15-PGDH) in triple-negative breast cancer. Breast Cancer Basic Clin Res.

[3] Jiang J, Dingledine R (2013). Prostaglandin receptor EP2 in the crosshairs of anti-inflammation, anti-cancer, and neuroprotection. Trends Pharmacol Sci.

[4] Kawahara K, Hohjoh H, Inazumi T, Tsuchiya S, Sugimoto Y (2015). Prostaglandin E2-induced inflammation: relevance of prostaglandin E receptors. Biochim Biophys Acta.

[5] Efsen E, Bonacchi A, Pastacaldi S, Valente AJ, Wenzel UO, Tosti-Guerra C (2001). Agonist-specific regulation of monocyte chemoattractant protein-1 expression by cyclooxygenase metabolites in hepatic stellate cells. Hepatology.

[6] Gallois C, Habib A, Tao J, Moulin S, Maclouf J, Mallat A (1998). Role of NF-kappaB in the antiproliferative effect of endothelin-1 and tumor necrosis factor-alpha in human hepatic stellate cells. Involvement of cyclooxygenase-2. J Biol Chem.

[7] Cheng J, Imanishi H, Liu W, Iwasaki A, Ueki N, Nakamura H (2002). Inhibition of the expression of alpha-smooth muscle actin in human hepatic stellate cell line, LI90, by a selective cyclooxygenase 2 inhibitor, NS-398. Biochem Biophys Res Commun.

[8] Planaguma A, Claria J, Miquel R, Lopez-Parra M, Titos E, Masferrer JL (2005). The selective cyclooxygenase-2 inhibitor SC-236 reduces liver fibrosis by mechanisms involving non-parenchymal cell apoptosis and PPARgamma activation. FASEB J.

[9] Gao JH, Wen SL, Tong H, Wang CH, Yang WJ, Tang SH (2016). Inhibition of cyclooxygenase-2 alleviates liver cirrhosis via improvement of the dysfunctional gut-liver axis in rats. Am J Physiol Gastrointest Liver Physiol.

[10] Paik YH, Kim JK, Lee JI, Kang SH, Kim DY, An SH (2009). Celecoxib induces hepatic stellate cell apoptosis through inhibition of Akt activation and suppresses hepatic fibrosis in rats. Gut.

[11] Yamamoto H, Kondo M, Nakamori S, Nagano H, Wakasa K, Sugita Y (2003). JTE-522, a cyclooxygenase-2 inhibitor, is an effective chemopreventive agent against rat experimental liver fibrosis1. Gastroenterology.

[12] O'Brien AJ, Fullerton JN, Massey KA, Auld G, Sewell G, James S (2014). Immunosuppression in acutely decompensated cirrhosis is mediated by prostaglandin E2. Nat Med.

[13] Penke LR, Huang SK, White ES, Peters-Golden M (2014). Prostaglandin E2 inhibits alpha-smooth muscle actin transcription during myofibroblast differentiation via distinct mechanisms of modulation of serum response factor and myocardin-related transcription factor-A. J Biol Chem.

[14] Mohamed R, Jayakumar C, Ramesh G (2013). Chronic administration of EP4-selective agonist exacerbates albuminuria and fibrosis of the kidney in streptozotocin-induced diabetic mice through IL-6. Lab Investig.

[15] Hui AY, Dannenberg AJ, Sung JJ, Subbaramaiah K, Du B, Olinga P (2004). Prostaglandin E2 inhibits transforming growth factor beta 1-mediated induction of collagen alpha 1(I) in hepatic stellate cells. J Hepatol.

[16] Brea R, Motino O, Frances D, Garcia-Monzon C, Vargas J, Fernandez-Velasco M (2018). PGE2 induces apoptosis of hepatic stellate cells and attenuates liver fibrosis in mice by downregulating miR-23a-5p and miR-28a-5p. Biochim Biophys Acta Mol Basis Dis.

[17] Puche JE, Saiman Y, Friedman SL (2013). Hepatic stellate cells and liver fibrosis. Compr Physiol.

[18] Tsuchida T, Friedman SL (2017). Mechanisms of hepatic stellate cell activation. Nat Rev Gastroenterol Hepatol.

[19] Tang J, Zhu X, Zhao J, Fung M, Li Y, Gao Z (2015). Tissue transglutaminase-regulated transformed growth factor-β1 in the parasite links *Schistosoma japonicum* infection with liver fibrosis. Mediat Inflamm.

[20] Seki E, Schwabe RF (2015). Hepatic inflammation and fibrosis: functional links and key pathways. Hepatology.

[21] Aoyama T, Paik YH, Seki E (2010). Toll-like receptor signaling and liver fibrosis. Gastroenterol Res Pract.

[22] Seki E, De Minicis S, Osterreicher CH, Kluwe J, Osawa Y, Brenner DA (2007). TLR4 enhances TGF-beta signaling and hepatic fibrosis. Nat Med.

[23] Liu C, Chen X, Yang L, Kisseleva T, Brenner DA, Seki E (2014). Transcriptional repression of the transforming growth factor beta (TGF-beta) pseudoreceptor BMP and activin membrane-bound inhibitor (BAMBI) by nuclear factor kappaB (NF-kappaB) p50 enhances TGF-beta signaling in hepatic stellate cells. J Biol Chem.

[24] Roderburg C, Urban GW, Bettermann K, Vucur M, Zimmermann H, Schmidt S (2011). Micro-RNA profiling reveals a role for miR-29 in human and murine liver fibrosis. Hepatology.

[25] Seki E, De Minicis S, Gwak GY, Kluwe J, Inokuchi S, Bursill CA (2009). CCR1 and CCR5 promote hepatic fibrosis in mice. J Clin Investig.

[26] Zhang Z, Lin C, Peng L, Ouyang Y, Cao Y, Wang J (2012). High mobility group box 1 activates Toll like receptor 4 signaling in hepatic stellate cells. Life Sci.

[27] Wen Z, Ji X, Tang J, Lin G, Xiao L, Liang C (2017). Positive feedback regulation between transglutaminase 2 and Toll-like receptor 4 signaling in hepatic stellate cells correlates with liver fibrosis post *Schistosoma japonicum* infection. Front Immunol.

[28] Zhang Y, Igwe OJ (2018). Lipopolysaccharide (LPS)-mediated priming of toll-like receptor 4 enhances oxidant-induced prostaglandin E2 biosynthesis in primary murine macrophages. Int Immunopharmacol.

[29] Samuchiwal SK, Balestrieri B, Raff H, Boyce JA (2017). Endogenous prostaglandin E2 amplifies IL-33 production by macrophages through an E prostanoid (EP)2/EP4-cAMP-EPAC-dependent pathway. J Biol Chem.

[30] Verbeek RE, Siersema PD, Ten Kate FJ, Fluiter K, Souza RF, Vleggaar FP (2014). Toll-like receptor 4 activation in Barrett’s esophagus results in a strong increase in COX-2 expression. J Gastroenterol.

[31] Tanigawa T, Odkhuu E, Morikawa A, Hayashi K, Sato T, Shibata R (2014). Immunological role of prostaglandin E2 production in mouse auditory cells in response to LPS. Innate Immun.

[32] Tang J, Huang H, Ji X, Zhu X, Li Y, She M (2014). Involvement of IL-13 and tissue transglutaminase in liver granuloma and fibrosis after *Schistosoma japonicum* infection. Mediat Inflamm.

[33] Zhao Y, Yang S, Li B, Li W, Wang J, Chen Z (2019). Alterations of the mice gut microbiome via *Schistosoma japonicum* ova-induced granuloma. Front Microbiol.

[34] Fukata M, Chen A, Klepper A, Krishnareddy S, Vamadevan AS, Thomas LS (2006). Cox-2 is regulated by Toll-like receptor-4 (TLR4) signaling: role in proliferation and apoptosis in the intestine. Gastroenterology.

[35] Chen CC (2006). Signal transduction pathways of inflammatory gene expressions and therapeutic implications. Curr Pharm Des.

[36] Brea R, Motiño O, Francés D, García-Monzón C, Vargas J, Fernández-Velasco M (2018). PGE(2) induces apoptosis of hepatic stellate cells and attenuates liver fibrosis in mice by downregulating miR-23a-5p and miR-28a-5p. Biochim Biophys Acta.

[37] Schippers M, Beljaars L, Post E, Lotersztajn S, Reker-Smit C, Han B (2017). Upregulation of Epac-1 in hepatic stellate cells by prostaglandin E(2) in liver fibrosis is associated with reduced fibrogenesis. J Pharmacol Exp Ther.

[38] Zhang Y, Igwe OJ (2018). Lipopolysaccharide (LPS)-mediated priming of toll-like receptor 4 enhances oxidant-induced prostaglandin E(2) biosynthesis in primary murine macrophages. Int Immunopharmacol.

[39] Samuchiwal SK, Balestrieri B, Raff H, Boyce JA (2017). Endogenous prostaglandin E(2) amplifies IL-33 production by macrophages through an E prostanoid (EP)(2)/EP(4)-cAMP-EPAC-dependent pathway. J Biol Chem.

